# Supplementary material for: Heat adaptation of phage T7 under an extended genetic code
Source: Virus Evol. 2021 Dec 1;7(2):veab100. doi: 10.1093/ve/veab100 (PMC8923235; doi:10.1093/ve/veab100)
Supplement: veab100_Supp [file veab100_supp.zip › Sequencing_Analysis.zip!/Sequencing_Analysis/S1/evidence/RA_33.html]

BRESEQ :: Evidence


| Read alignment evidence... | | | | | | | | | | | |
| --- | --- | --- | --- | --- | --- | --- | --- | --- | --- | --- | --- |
|  | seq id | position | | ref | new | freq | score (cons/poly) | reads | annotation | genes | product |
| \* | NC\_001604.1 | 35,366 | 0 | C | A | 77.2% | 5753.9 / inf | 4301 | intergenic (–/–) | *–/–* | –/– |
| Reads supporting (aligned to +/- strand):  **ref** base C (268/209);  **major** base A (1601/1343);  **minor** base T (471/399);  **total** (2342/1959) | | | | | | | | | | | |
| Fisher's exact test for biased strand distribution *p*-value = 9.08e-01 | | | | | | | | | | | |
| Kolmogorov-Smirnov test that lower quality scores support variant *p*-value = 5.11e-01 | | | | | | | | | | | |

Only 100 of 4312 total aligned reads displayed.  

`CTGTTCCGTTTGGTCAACTAAAGACCATGAACCAGAACTCATGGCAAGCACGTAATGAAGCCTTACAGTTCCGTAATGAGGCTGAGACTTTCAGAAACCAAGCGGAGGGCTTTAAGAACGAGTCCAGTACCAACGCTACGAACACAAAGCAGTGGCGCGATGAGACCAAGGGTTTCCGAGACGAAGCCAAGCGGTTCAAGAATACGGCTGGTCAATACGCTACATCTGCTGGGAACTCTGCTTCCGCTGCGCATCAATCTGAGGTAAACGCTGAGAACTCTGCCACAGCATCCGCTAACTCTGCTCATTTGGCAGAACAGCAAGCAGACCGTGCGGAACGTGAGGCAGACAAGCTGGAAAATTACAATGGATTGGCTGGTGCAATTGATAAGGTAGATGGAACCAATGTGTACTGGAAAGGAAATATTCACGCTAACGGGCGCCTTTACATGACCACAAACGGTTTTGACTGTGGCCAGTATCAACAGTTCTTTGGTGGTGTCACTAATCGTTACTCTGTCATGGAGTGGGGAGATGAGAACGGATGGCTGATGTATGTTCAACGTAGAGAGTGGACAACAGCGATAGGCGGTAACATCC  >  NC_001604.1/35066‑35665`  

                                                                                                                                                                                                                                                                                                            `|`                                                                                                                                                                                                                                                                                                             
    `tCCGTTTGGTCAACTAAAGACCATGGACCAGAACTCATGGCAAGCACGTAATGAAGCCTTACAGTTCCGTAATGAGGCTGAGACTTTCAGAAACCAAGCGGAGGGCTTTAAGAACGAGTCCAGTACCAACGCTACGAACACAAAGCAGTGGCGCGATGAGACCAAGGGTTTCCGAGACGAAGCCAAGCGGTTCAAGAATACGGCTGGTCAATACGCTACATCTGCTGGGAACTCTGCTTCCGCTGCGCATCAATCTGAGGTAAACGCTGAGAACTCTGCCACAGCATCCGCTAACTATGCt                                                                                                                                                                                                                                                                                                         <  1:511173/301‑1 (MQ=255)`  
    `tCCGATTGGTCAACTAAAGGTCATGGACCAAAACTCATGGCAAGCACGTAATGAAGCCTTACAGTGCCGTAATGAGGCTGAGACTTTCAGAAACCAAGCGGAGGGCGTTAAGAACGAGTCCAGTACCAACGCTACGAACACAAAGCAGTGGCGCGATGAGACCAAGGGTTTCCGAGACGAAGCCAAGCGGTTCAAGAATACGGCTGGTCAATACGCTACATCTGCTGGGAACTCTGCTTCCGCTGCGCATCAATCTGAGGTAAACGCTGAGAACTCTGCCACAGCATCCGCTAACTATGCt                                                                                                                                                                                                                                                                                                         <  1:19247/301‑1 (MQ=255)`  
     `ccGTTTGGTCAACTAAAGACCATGGACCAGAACTCATTGCAAGCACGTAATGAAGCCTTACAGTTCCGTAATGAGGCTGAGACTTTCAGAAACCAAGCGGAGGGCTTTAAGAACGAGTCCAGTACCAACGCTACGAACACAAAGCATTGGCGCGATGAGACCAAGGGTTTCCGAGACGAAGCCAAGCGGTTCAAGAATATGGCTGGTCAATACGCTACATCTGCTGGGAACTCTGCTTCCGCTGCGCATCAATCTGAGGTAAACGCTGAGAACTCTGCCACAGCATCCGCTAACTATGCTc                                                                                                                                                                                                                                                                                                        <  1:226822/301‑1 (MQ=255)`  
       `aagagacagaCTAAAGACCATGGACCAGAACTCATGGCAAGCACGTAATGAAGCCTTACAGTTCCGTAATGAGGCTGAGACTTTCAGAAACCAAGCGGAGGGCTTTAAGAACGAGTCCAGTACCAACGCTACGAACACAAAGCAGTGGCGCGATGAGACCAAGGGTTTCCGAGACGAAGCCAAGCGGTTCAAGGATACGGCTGGTCAATACGCTACATCTGCTGGGAACTCTGCTTCCGCTGCGCATCAATCTGAGGTAAACGCTGAGAACTCTGCCACAGCATCCGCTAACTATGCTCAt                                                                                                                                                                                                                                                                                                      <  1:544951/292‑1 (MQ=255)`  
            `acagaCTAAAGACCATGGACCAGAACTCATGGCAAGCACGTAATGAAGCCTTACAGTTCCGTAATGAGGCTGAGACTTTCAGAAACCAAGCGGAGGGCTTTAAGAACGAGTCCAGTACCAACGCTACGAACACAAAGCAGTGGCGCGATGAGACCAAGGGTTTCCGAGACGAAGCCGAGCGGTTCAAGAATACGGCTGGTCAATACGCTACATCTGCTGGGAACTCTGCTTCCGCTGCGCATCAATCTGAGGTAAACGCTGAGAACTCTGCCACAGCATCCGCTAACTTTGCTCATTTGGc                                                                                                                                                                                                                                                                                                 <  1:198191/297‑1 (MQ=255)`  
             `ccAACTAAAGACCATGGACCAGAACTAATGGCAAGCACGTAATGAAGCCTTACAGTTCCGTAATGAGGCTGAGACTTTCAGAAACCAAGCGGAGGGCTTTAAGAACGAGTCCAGTACCAACGCTACGAACAAAAAGCAGTGGCGCGATGAGACCAAGGGTTTCCGAGACGAAGCCAAGCGGTTCAAGAATACGGCTGGTCAATACGCTACATCTGCTGGGAACTCTGCTTCCGCTGCGCATCAATCTGAGGTAAACGCTGAGAACTCTGCCACAGCATCCGCTAACTATGCTCATTTGGCa                                                                                                                                                                                                                                                                                                <  1:442347/300‑1 (MQ=255)`  
                `aCTAAAGACCATGGACCAGAACTCATGGCAAGCACGTAATGAAGCCTTACAGTTCCGTAATGAAGCTGAGACTTTCAGAAACCAAGCGGAGGGCTTTAAGAACGAGTCCAGTACCAACGCTACGAACACAAAGCAGTGGCGCGATGAGACAAAGGGTTTCCGCGACGAAGCCAAGCGGTTCAAGGATACGGCTGGTCAATACGCTACATCTGCTGGGAACTCTGCTTCCGCTGCGCATCAATCTGAGGTAAACGCTGAGAACTCTGCCACAGCATCCGCTAACTTTGCTCATTTGGCAGaa                                                                                                                                                                                                                                                                                             >  1:494153/1‑301 (MQ=255)`  
                   `aaaGACCAGGGACAAGAACTCATGGAAAGCACGTAATGAAGCCTTACAGTTCCGGAATGAGGCTGAGACTTTCAGAAACCAAGCGGAGGGCTTGAAGAACGAGTCCAGTACCAACGCTACGAACACAAAGCAGTGGCGCGATGAGACCAAGGGTTTCCGAGACGAAGCCAAGCGGTTCAAGAATACGGCTGGTCAATACGCTACATCTGCTGGGAACTCTGCTTCCGCTGCGCATCAATCTGAGGTAAACGCTGAGAACTCTGCCACAGCATCCGCTAACTATGCTCATTTGGCAGAACag                                                                                                                                                                                                                                                                                          <  1:637873/301‑1 (MQ=255)`  
                       `aCCATGGACCAGAACTCATGGCAAGCACGTAATGAAGCCTTACAGTTCCGTAATGAGGCTGAGACTTTCAGAAACCAAGCGGAGGGCTTTAAGAACGAGTCCAGTACCAACGCTACGAACACAAAGCAGTGGCGCGATGAGACCAAGGGTTTCCGAGACGAAGCCAAGCGGTTCAAGAATACGGCTGGTCAATACGCTACATCTGCTGGGAACTCTGCTTCCGCTGCGCATCAATCTGAGGTAAACGCTGAGAACTCTGCCCCAGCATCCGCTAACTATGCTCATTTGGCAGAACagcaaa                                                                                                                                                                                                                                                                                      >  1:100149/1‑300 (MQ=255)`  
                        `acATGGACAAGAACTAATGGCAAGAACGTAATGAAGCCTACCAGTTCCGTAATGAGGCTGAGACTGTCAGAAACCAAGCGGAGGGCTTTAAGAACGAGTCCAGTAGCAACGCTACGAACACAAAGCAGGGGCACGATGAGACCAAGGGTTTCCGAGACGAAGCCAAGCGGTTCAAGAAGACGGCTGGTCAATACGCTACATCTGCTGGGATCTCTGCTTCCGCTGCTCATCAATCTGAGGTAAACGCTGAGAACTCTGCCACAGCATCCGCTAACTTTGCTCAGTTGGCAGAACagcaagc                                                                                                                                                                                                                                                                                     <  1:632168/300‑1 (MQ=255)`  
                          `agggaCCCGAACTCAGGGCAAGCACGTAATGAAGCCTTACATTTCCGTAATGAGGCTGAGACTTTCAGAAACCAAGCGGAGTGCTTTAAGAACGAGTCCAGTACCAACGCTACGAACACAAAGCAGTGGCGCGATGAGACAAAGGGTTTCCGAGACGAAGCCAAGCGGTTCAAGGATACGGCTGGTCAATACGCTACATCTGCTGGGAACTCTGCTTCCGCTGCGCATCAATCTGAGGTAAACGCTGAGAACTATGCCACAGCATCCGCTAACTCTGCTCATTTGGCAGAACAGCAAGCAg                                                                                                                                                                                                                                                                                   <  1:220357/297‑1 (MQ=255)`  
                            `ggacaaGAACTCATGGCAAGCACGTAATGAAGCCTTACAGTTCCGTAATGAGGCTGAGACTTTCAGAAACCAAGCGGAGGGCTTTAAGAACGAGTCCAGTACCAACGCTACGAACACAAAGCAGTGGCGCGATGAGACCAAGGGTTTCCGAGACGAAGCCGAGCGGTTCAAGAATACGGCTGGTCAATACGCTACATCTGCTGGGAACTCTGCTTCCGCTGCGCATCAATCTGAGGTAAACGCTGAGAACTCTGCCACAGCATCCGCTAACTATGCTCATTTGGCAGAACAGCAAGCAGAc                                                                                                                                                                                                                                                                                 <  1:312485/296‑1 (MQ=255)`  
                            `agatgtgtataagagacaggaacatAATGAAGCCTTAAAGGACCGGAATGGGGCGGAGACGTTCAGAAACCAAGCGGAGGGCTTTAAGAACGCGTACAGTACAAACGCTACGAACACAAAGCAGTGGCGCGATGAGACCAAGGGTTTCCGAGACGAAGCCAAGCGGTTAAAGAATACGGCTGGTCAATACGCTACATCTGCTGGGAACTCTGCTTCCGCTGCGCATCAATCTGGGGTAAACGCTGAGAACGCTGCCACAGCATCCGCTAACTATGCTCATTTGGCAGAACAGCAAGCAGAc                                                                                                                                                                                                                                                                                 <  1:381611/277‑1 (MQ=255)`  
                                  `gAACTCATGGCAAGCACGTAATGAAGCCTTACAGTTCCGTAATGAGGCTGAGACTTTCAGAAACCAAGCGGAGGGCTTTACGAACGAGTCCAGTACCAACTCTACGCACACAAAGCAGTGGCGCGATGAGACCAATGGTTTCCGAGATGAAGCTAAGCGTCTCCAGGATCCGGCTGGTCAATACGCTACATCTGCCGGGAACTCTGCTTCCGCTTCGCCTTAATCTGTGGTAAACGCTGAGAACTTTGCCACTGCATCCGCTCACTCTGCTCATTTGTCAGAACAGCAAGCAGACCGTGct                                                                                                                                                                                                                                                                           >  1:403572/1‑300 (MQ=255)`  
                                  `gAACTCATGGCAAGCACGTAATGAAGCCTTACAGTTCCGTAATGAGGCTGAGACTTTCAGAAACCAAGCGGAGGGCTTTAAGAACGAGTCCAGTACCAACGCTACGAACACAAAGCAGTGGCGCGATGAGACCAAGGGTTTCCGAGACGAAGCCAAGCGGTTCAAGAATATGGCTGGTCAATACGCTACATCTGCTGGGAACTCTGCTTCCGCTGCGCATCAATCTGAGGTAAACGCTGAGAACTCTGCCACAGCATCCGCTAACTATGCTCATTTGGCAGAACAGCAAGCAGACCGTGCg                                                                                                                                                                                                                                                                           >  1:395154/1‑301 (MQ=255)`  
                                         `tGGCAAGCACGTAATGAAGCCTTACAGTTCCGTAATGAGGCTGAGACTTTCAGAAACCAAGCGGAGGGCTTTAAGAACGAGTCCAGTACCAACGCTACGAACACAAAGCAGTGGCGCGATGAGACCAAGGGTTTCCGAGACGAAGCCAAGCGGTTCAAGGATACGGCTGGTCAATACGCTACATCTGCTGGGAACTCTGCTTCCGCTGCGCATCAATCTGAGGTAAACGCTGAGAACTCTGCCACAGCATCCGCTAACTTTGCTCATTTGGCAGAACAGCAAGCAGACCGTGCGGAACGTg                                                                                                                                                                                                                                                                    >  1:7361/1‑301 (MQ=255)`  
                                            `aaaGCACGGAAGGAAGCCTTACAGTTCCGTAATGAGGATGAGACTTTCAGAAACCAAGCGGAGGGCTTTAAGAAAGATTCCAGTACCAACGCTACGAACACAAAGCAGTGGCGCGATGAGACCAAGGGTTTCCGAGACGAAGCCAAGCGGTTCAAGGATACGGCTTGTCAATACGCGACATCTGCTGGGAACTCTGCTTCCGCTGCGCATCAATCTGAGGT‑AACGCTGAGAACTCTGCCACAGCATCCGCTAACTTTGCTCATTTGGCAGAACAGCAAGCAGACCGTGCGGAACGTGAGGc                                                                                                                                                                                                                                                                <  1:274735/300‑1 (MQ=255)`  
                                               `aataagagacaggCCTTACAGTTCCGTAATGAGGCTGAGACTTTCAGAAACCAGGCGGAGGGCTTTAAGAACGAGTCCAGTACCAGCGCTACGAACACAAAGCAGTGGCGCGATGAGACCAAGGGTTTCCGAGACGAAGCCAAGCGGTTCAAGAATACGGCTGGTCAATACGCTACATCTGCTGGGAACTCTGCTTCCGCTGCGCATCAATCTGAGGTAAACGCTGAGAACTCTGCCACAGCATCCGCTAACTATGCTCATTTGGCAGAACAGCAAGCAGACCGTGCGGAACGTGAGGCAg                                                                                                                                                                                                                                                              <  1:203549/289‑1 (MQ=255)`  
                                                    `tAATGAAGCCTTACAGTTCCGTAATGAGGCTGAGACTTTCAGAAACCAAGCGGAGGGATTTAAGAACGAGTCCAGTACCAACGCTACGAACACAAAGCAGTGGCGCGATGAGACCAAGGGTTTCCTAGACGAAGCCAAGCGGTTCAAGTATACGGCTGGTAAATACGCTACATCTGCTGGGAACTCTGCTTCCGCTGAGCATCAATCTGAGGTAAACGCTGAGAACTCTGCCACAGCATCCGCTAACTATGAACATTTGACAGAACAGAAAGCAGACCGTACGGAACGTGAGGCAGGCAAg                                                                                                                                                                                                                                                         >  1:93259/1‑301 (MQ=255)`  
                                                    `tAATGAAGCCTTACAGTTCCGTAATGAGGCTGAGACTTTAAGAAACCAAGCGGAGGGCTTTAAGAACGAGTCCAGTACCAACGCTACGAACACAAAGCAGTGGAGCGATGAGACCAAGGGTTTCCGAGACGAAGCCAAGCGGTTCAAGGATACGGCTGGTCAATACGCTACATCTGCTGGGAACTCTGCTTCCGCTGCGCATCAATCTGAGGTAAACGCTGAGAACTATGCCACAGCATCCGCTAACTCTGCTCATTTGGCAGAACAGCAAGCAGACCGTGCGGAACGTGAGGCAGACAAg                                                                                                                                                                                                                                                         >  1:522388/1‑301 (MQ=255)`  
                                                        `gAAGCCTTACAGTTCCGTAATGAGGCTGAGACTTTCAGAAACCAAGCGGAGGGCTTTAAGAACGAGTCCAGTACCAACGCTACGAACACAAAGCAGTGGCGCGATGAGACCAAGGGTTTCCGAGACGAAGCCAAGCGGTTCAAGGATACGGCTGGTCAATACGCTACATCTGCTGGGAACTCTGCTTCCGCTGCGCATCAATCTGAGGTAAACGCTGAGAACTCTGCCACAGCATCCGCTAACTATGCTCATTTGGCAGAACAGCAAGCAGACCGTGCGGAACGTGAGGCAGACAAGCTgg                                                                                                                                                                                                                                                     >  1:250235/1‑301 (MQ=255)`  
                                                         `aaGCCTTACAGTTCCGTAATGAGGCTGAGACTTTCAGAAACCAAGCGGAGGGCTTTAAGAACGAGTCCAGTACCAACGCTACGAACACAAAGCAGTGGCGCGATGAGACCAAGGGTTTCCGAGACGAAGCCAAGCGGTTCAAGAATACGGCTGGTCAATACGCTACATCTGCTGGGAACTCTGCTTCCGCTGCGCATCAATCTGAGGTAAACGCTGAGAACTCTGCCACAGCATCCGCTAATTATGCTCATTTGGCAGAACAGCAAGCAGACCGTGCGGAACGTGAGGCAGACAAGCTGGa                                                                                                                                                                                                                                                    >  1:594829/1‑301 (MQ=255)`  
                                                           `gCCTTACAGTTCCGTAATGAGGCTGAGACTTTCAGAAACCAAGCGGAGGGCTTTAAGAACGAGTCCAGTACCAACGCTACGAACACAAAGCAGTGGCGCGATGAGACCACGGGTTTCCGAGACGAAGCCGAGCGGTTCAAGAATACGGCTGGTCAATACGCTACATCTGCTGGGAACTCTGCTTCCGCTGCGCATCAATCTGAGGTAAACGCTGAGAACGCTGCCACAGCATCCGCTAACTCTGCTCATTTGGCGGAACAGCAAGCAGACCGTGCGGTACGTGAGGCAGACAAGCTGGaaa                                                                                                                                                                                                                                                  >  1:79775/1‑301 (MQ=255)`  
                                                           `gCCTTACAGTTCCGTAATGAGGCTGAGACTTTCAGAAACCAAGCGGAGGGCTTTAAGAACGAGTCCAGTACCAACGCTACGAACACAAAGCAGTGGCGCGATGAGACCAAGGGTTTCCGAGACGAAGCCAAGCGGTTCAAGGATACGGCTGGTCAATACGCTACATCTGCTGTGAACTCTGCTTCCGCTGCGCATCAATCTGAGGTAAACGCTGAGAACTCTGCCACAGCATCCGCTAACTTTGCTCATTTGGCAGAACAGCAAGCAGACCGTGCGGAACGTGAGTCAGACAAGCTGGaaa                                                                                                                                                                                                                                                  >  1:612276/1‑301 (MQ=255)`  
                                                                  `aGTTCCGTAATGAGGCTGAGACTTTCAGAAACCAAGCGGAGGGCTTTAAGAACGAGTCCAGTACCAACGCTACGAACACAAAGCAGTGGCGCGATGAGACCAAGGGTTTCCGAGACGAAGCCAAGCGGTTCAAGGATACGGCTGGTCAATACGCTACATCTGCTGGGAACTCTGCTTCCGCTGCGCATCAATCTGATGTAAACGCTGAGAACTCTGCCACAGCATCCGCTAACTCTGCTCATTTGGCAGAACAGCAAGCAGACCGTGCGGAACGTGAGGCAGACAAGCTGGAAAATTACaa                                                                                                                                                                                                                                           <  1:125797/301‑1 (MQ=255)`  
                                                                     `tCCGTAATGAGGCTGAGACTTTCAGAAACCAAGCGGAGGGCTTTAAGAACGAGTCCAGTACCAACGCTACGAACACAAACCAGTGGCGCGATGAGACCAAGGGTTTCCGAGACGAAGCCGAGCGGTTCTAGAATACGGCTGGTACATACGCTACATCTGCTGGTAACTCTGCTTCCGCTGCGCATCCATCTGAGGTAAACGCTGAGAACTCTGCCACAGCATCCGCTAACTATGCTCATTTGGCAGAACAGCAAGCAGACCGTGCGGTACGTGAGGCAGACAAGCTGGaaactgtctcttt                                                                                                                                                                                                                                        >  1:580135/1‑291 (MQ=255)`  
                                                                            `tGAGGCTGAGACTTTCAGAAACCAAGCGGAGGGCTTTAAGAACGAGTCCAGTACCAACGCTACGAACACAAAGCAGTGGCGCGATGAGACCAAGGGTTTCCGAGACGAAGCCAAGCGGTTCAAGAATACGGCTGGTCAATACGCTACATCTGCTGGGAACTCTGCTTCCGCTGCGCATCAATCTGAGGTAAACGCTGAGAACTCTGCCACAGCATCCGCTAACTATGCTCATTTGGCAGAACAGCAAGCAGACCGTGCGGAACGTGAGGCAGACAAGCTGGAAAATTACAATGGATTGGCt                                                                                                                                                                                                                                 <  1:500811/301‑1 (MQ=255)`  
                                                                               `ggCTGAGACTTTCAGAAACCAATCGGAGGGCTTTAAGAACGAGTCCAGTACCAACGCTACGAACACAAAGCAGTGGCGCGATGAGACCAAGGGTTTCCGAGACGAAGCCAAGCGGTTCAAGAATACGGCTGGTCAATACGCTACATCTGCTAGGAACTCTGCTTCCGCTGCGCATCAATCTGAGGTAAACGCTGAGAACTCTGCCACAGCATCCGCTAACTATGCTCATTTGGCAGAACAGCAAGCAGACCGTGCGGAACGTGAGGCAGACAAGCTGGAAAATTACAATGGATTGGcctgt                                                                                                                                                                                                                              >  1:186919/1‑297 (MQ=255)`  
                                                                               `ggCTGAGACTTTCAGAAACCAAGCGGAGGGCTTTAAGAACGAGTCCAGTACCAACGCTACGAACACAAAGCAGTGGCGCGATGAGACCAAGGGTTTCCGAGACGAAGCCAAGCGGTTCAAGAATACGGCTGGTCAATACGCTACATCTGCTGGGAACTCTGCTTCCGCTGCGCATCAATCTGAGGTAAACGCTGAGAACTCTGCCACAGCATCCGCTAACTATGCTCATTTGGCAGAACAGCAAGCAGACCGTGCGGAACGTGAGGCAGACAAGCTGGAAAATTACAATGGATTGGCTGGt                                                                                                                                                                                                                              >  1:10512/1‑301 (MQ=255)`  
                                                                                      `aCTTTCAGAAACCAATCGGAGGGCTTTAAGAACGGGTCCAGTACCAACGCTACGAACACAAAGCAGTGGCGCGATGAGACCAAGGGTTTACTCGACTAAGCCAAGCGGTTCAAGAATACGGCTGGTCAATACGCTACATCTGCTAGGATCTCTGCTTCCGCTGCGCATCAATCGGAGGTAAACGCTGAGAACTCTGCGACAGCATCCGCTAACTTTGCTCATTTGGCAGAACAGCAAGCAGACCGTGCGTAACGTGAGGCAGACAAGCTGGAAAATTACAATGGATTGGCTGGTGCAATTg                                                                                                                                                                                                                       <  1:319522/301‑1 (MQ=255)`  
                                                                                        `tttCAGAAACCAAGCGGAGGGCTTTAAGAACGAGTCCAGTACCAACGCTACGAACACAAAGCAGTGGCGCGATAAGACCAAGGGTTTCCGAGACGAAGCCAAGCGGCTCAAGAATACGGCTGGTCAATACGCTACATCTGCTGGGAACTCTGCTTCCGCTGCGCATCAATCTGAGGTAAACGCTGAGAACTATGCCACAGCATCCGCTAACTATGCTCATTTGGCAGAACAGCAAGCAGACCGTGCGGAACGTGAGGCAGACAAGCTGGAAAATTACAATGGATTGGCTGGTGCAAATgct                                                                                                                                                                                                                     >  1:527231/1‑299 (MQ=255)`  
                                                                                             `aaaaaCAAGGGGAGGGCTTTAAGAACGAGTCCAGTAACAACGCTACGAAAACAAAGCAGGGGCGCGATGAGAACAAGGGTTTCCGAGACGAAGCCAAGCGGTTCAAGGATACGGCTGGTCAATACGCGACATCTGCTGGGAACTCTGCTTCCGCTGCGCATCAATCTGAGGTAAACGCTGAGAACTCTGCCACAGAATCCGCTAACTTTGCCCATTTGGCAGAACAGCAAGCAGACCGTGCGGAACGTGAGGCAGACAAGCTGGAAAATTACAATGGATTGGCTGGTGCAATTGATAAGGt                                                                                                                                                                                                                <  1:244484/300‑1 (MQ=255)`  
                                                                                             `aaaaCCAAGCGGAGGGCTTTAAGAACGAGTCCAGTACCAACGCTAAGAACACAAAGCAGTGGCGCGATGAGACCAAGGGTTTCCGAGACGAAGCCAAGCGGTTCAAGAATACGGCTGGTCAATACGCTACATCTGCTTGGAACTCTGCTTCCGCTGCGCATCAATCTGAGGTAAACGCTGAGAACTTTGCCACAGCATCCGCTAACTATGCTCATTTGGCAGAACAGCAAGCAGACCGTGCGGAACGTGAGGCAGACAAGCTGGAAAATTACAATGGATTGGCTGGTGCAATGGATAAGGt                                                                                                                                                                                                                <  1:298875/300‑1 (MQ=255)`  
                                                                                                   `acgCGGAGGGCTTTAAGAACGAGTCCAGTACCAACGCTACGAACACAAAGCAGTGGCGCGATGAGACCAAGGGTTTCCGAGACGAAGCCAAGCGGTTCAAGAATACGGCTGGTCAATACGCTACATCTGCTGCGAACTCTGCTTCCGCTGCGCATCAATCTGAGGTAAACGCTGAGAACTCTGCCACAGCATCCGCTAACTATGCTCATTTGGCAGAACAGCAAGCAGACCGTGCGGCACGTGAGGCAGACAAGCTGGAAAATTACAATGGATTGGCTGGTGCAATTGATAAGGTAGATgg                                                                                                                                                                                                          <  1:604912/299‑1 (MQ=255)`  
                                                                                                   `aaGCGGGTGGCTTTAAGAACTAGTCAAGTACCAACGATACGAACACAAAGCAGTGGCGCGATGAGACCAAGGGTTTCCGAGACGAAGCCAAGCGGTTCAAGGATACGGCTGGTCAATACGCTACATCTGCTGGGAACTCTGCTTCCGCTGAGCATCAATCGGAGGTAAACGCTGAGAACTCTGCCACAGCATCCGCTAACTTTGCTCATTTGGCAGAACAGCAAGCAGACCGTGCGGAACGTGAGGCAGACAAGCTGGAAAATTACAATGGATTGGCTGGTGCAATTGATAAGGTAGATgg                                                                                                                                                                                                          <  1:149127/301‑1 (MQ=255)`  
                                                                                                     `gCGGAGGGCTTTAAGAACGAGTCCAGTACCAACGCTACGAACACAAAGCAGTGGCGCGATGAGACCAAGGGTTTCCGAGACGAAGCCGAGCGGTTCAAGAATACGGCTGGTCAATACGCTACATCTGCTGGGAACTCTGCTTCCGCTGCGCATCAATCTGAGGTAAACGCTGAGAACTCTGCCACAGCATCCGCTAACTATGCTCATTTGGCAGAACAGCAAGCAGACCGTGCGGAACGTGAGGCAGACAAGCTGGAAAATTACAATGGATTGGCTGGTGcctgtctcttatacacatctc                                                                                                                                                                                                        >  1:291350/1‑281 (MQ=255)`  
                                                                                                       `ggAAGGCGTTAAGCACGAGTCCAGTACCAACGCTACGAACACAAAGCAGTGGCGCGATGAGACCAAGGGTTTCCGAGACGAAGCCAAGCGGTTCAAGAATACGGCTGGTCAATACGCTACATCTGCTGGGAACTCTGCTACCGCTGCGCATCAATCTGAGGTAAACGCTGAGAACTCTGCCACAGCATCCGCTAACTATGCTCATTTGGCAGAACAGCAAGCAGACCGTGCGGAACGTGAGGCAGACAAGCTGGAAAATTACAATGGATTGGCTGGTGCAATTGATAAGGTAGATGGAAcc                                                                                                                                                                                                      <  1:143263/301‑1 (MQ=255)`  
                                                                                                          `gggCTTTAAGAACGAGTCCAGTACCAACGCTACGAACACAAAGCCGTCGCGCGATGAGACCAAGGGTTTCCGAGACGAAGCCGAGCGGTTCAAGAATACGGCTGGTCAATACGCTACATCTGCTGGGAACTCTGCTTCCGCTGCGCATCAATCTGAGGTAAACGCTGAGAACTCTGCCACAGCATCCGCTAACTATGCTCATTTGGCAGAACAGCAAGCAGACCGTGCGGAACGTGAGGCAGACAAGCTGGAAAATTACAATGGATTGGCTGGTGCAATTGATAAGGTAGATGGAACCAAt                                                                                                                                                                                                   >  1:341405/1‑301 (MQ=255)`  
                                                                                                           `gtcTTTAAGAACGAGTCCAGTACCCACGCTACGAACAACAAGCAGTGGCGCGATGAGACCAAGGGTTTCCGAGACGAAGCCAAGCGGTTCAAGGATACGGCTGGTCAATACGCTACATCTGCTGGGAACTCTGCTTCCGCTGCGCATCAATCTGAGGTAAACGCTGAGAACTCTGCCACAGCATCCGCTAACTTTGCTCATTTGGCAGAACAGCAAGCAGACCGTGCGGAACGTGAGGCAGACAAGCTGGAAAATTACAATGGATTGGCTGGTGCAATTGATAAGGTAGATGGAACCAAtg                                                                                                                                                                                                  <  1:325423/299‑1 (MQ=255)`  
                                                                                                           `ggCTTTAAGAACGAGTCCAGTACCAACGCTACGAACACAAAGCAGTGGCGCGATGAGACCAAGGGTTTCCGAGACGAAGCCAAGCGGTTCAAGAATACGGCTGGTCAATACGCTACATCTGCTGGGAACTCTGCTTCCGCTGCGCATCAATCTGAGGTAAACGCTGAGAACTCTGCCACAGCATCCGCTAACTATGCTCATTTGGCAGAACAGCAAGCAGACCATGCGGAACGTGAGGCAGACAAGCTGGAAAATTACAATGGATTGGCTGGTGCAATTGATAAGGTAGactgtctcttat                                                                                                                                                                                                  >  1:516891/1‑290 (MQ=255)`  
                                                                                                            `gCTTTAAGAACGAGTCCAGTACCAACGCTACGAACACAAAGCAGTGGCGCGATGAGACCAAGGGTTTCCGAGACGAAGCCAAGCGGTTCAAGAATATGGCTGGTCAATACGCTACATCTGCTGGGAACTCTGCTTCCGCTGCGCATCAATCTGAGGTAAACGCTGAGAACTCTGCCACAGCATCCGCTAACTATGCTCATTTGGCAGAACAGCAAGCAGACCGTGCGGAACGTTAGGCAGACAAGCTGGAACATTACAATGGATTGGCTGGTGCAATTGATAAGGTAGATGGAACCAAtgc                                                                                                                                                                                                 >  1:423443/1‑300 (MQ=255)`  
                                                                                                                   `gAACGAGACCAGTACCAACGCTACGAACACAAAGCAGTGGCGCGATGAGACCAAGGGTTTCCGAGACGAAGCCAAGCGGTTCAAGAATACGGCTGGTCAATACGCTACATCTGCTGGGAACTCTGCTTCCGCTGCGCATCAATCTGAGGTAAACGCTGAGAACTCTGCCACAGCATCCGCTAACTATGCTCATTTGGCAGAACAGCAAGCAGACCGTGCGGAACGTGAGGCAGACAAGCTGGAAAATTACAATGGATTGGCTGGTGCAATTGATAAGGTAGATGGAACCAATGTGTACTgg                                                                                                                                                                                          >  1:642894/1‑301 (MQ=255)`  
                                                                                                                    `aaCGAGTCCAGTACCAACGCTACTAACACAAAGCAGTGGCGCGATGAGACCAAGGGATTCCGAGACGAAGCCAAGCGGTTCAAGGATACGGCTGGTCAATACGCTACATCTGCTGGGAACTCTGCCTCCGCTGCGCATCAAACTGAGGTAAACGCTGAGAACTCTTCAACAGCATCCGCTAACTATGCCCATTTGGCAGAACAGAAAGCAGACCGTGCGGAACGTGAGGCAGACAAGCTGGAAAATTACAATGGATTGGCTGGTGCAATTGATAAAGTAAATGGAAccctctcctttatac                                                                                                                                                                                         >  1:232331/1‑288 (MQ=255)`  
                                                                                                                    `aaCGAGTCCAGTACCAACGCTACGAACACAAAGCAGTGGCGCGATGAGACCAAGGGTTTCCGAGACGAAGCCAAGCGGTTCAAGAATACGGCTGGTCAATACGCTACATCTGCTGGGAACTCTGCTTCCGCTGCGCATCAATCTGAGGTAAACGCTGAGAACTCTGCCACAGCATCCGCTAACTATGCTCATTTGGCAGAACAGCAAGCAGACCGTGCGGAACGTGAGGCAGACAAGCTGGAAAATTACAATGGATTGGATGGTGCAATTGATAAGGTAGATggctgtctcttatacaaat                                                                                                                                                                                         >  1:550347/1‑284 (MQ=255)`  
                                                                                                                         `gTCCAGTACCAACGCTACGAACACAAAGCAGTGGCGCGATGAGACCAAGGGTTTCCGAGACGAAGCCGAGCGGTTCAAGAATACGGCTGGTCAATACGCTACATCTGCTGGGAACTCTGCTTCCGCTGCGCATCAATCTGAGGTAAACGCTGAGAACGCTGCCACAGCATCCGCTAACTCTGCTCATTTGGCAGAACAGCAAGCAGACCGTGCGGAACGTGAGGCAGACAAGCTGGAAAATTACAATGTATTGGCTGGTGCAATTGATAAGGTAGATGGAACCAATGTGTACTggaaagga                                                                                                                                                                                    >  1:653491/1‑301 (MQ=255)`  
                                                                                                                         `gTCCAGTACCAACGCTACGAACACAAAGCAGTGGCGCGATGAGACCAAGGGTTTCCGAGACGAAGCCAAGCGGTTCAAGGATACGGCTGGTCAATACGCTACATCTGCTGGGAACTCTGCTTCCGCTGCGCATCAATCTGAGGTAAACGCTGAGAACTCTGCCACAGCATCCGCTAACTATGCTCATTTGGCAGAACAGCAAGCAGACCGTGCGGAACGTGAGGCAGACAAGCTGGAAAATTACAATGGATTGGCTGGTGCAATTGATAAGGTAGATGGAACCAATGTGTACTggaaagga                                                                                                                                                                                    >  1:285605/1‑301 (MQ=255)`  
                                                                                                                              `atACCAACGCTAAGAACAAAAAGCAGTGGCGCGATGAGACCAAGGTTTTCCGAGACGAATCCGAGCGGTTCAAGAATACGGCTGGTCAATACGCTACATCGGCTGGGAACTCTGCTTCCGCTGCGCATCAATCTGAGGTAAACGCTGAGAACGCTGCCACAGCATCCGCTAACTTTGCTCATTTGGCAGAACAGCAAGCAGACCGTGCGGAACGTGAGGCAGACAAGCTGGAAAATTACAATGGATTGGCTGGTGCAATTGATAAGGTAGATGGAACCAATGTGTACTGGAAAGGAAatat                                                                                                                                                                               <  1:346844/300‑1 (MQ=255)`  
                                                                                                                               `gaCCAAAGCTAAGGACACAAAGCAGTAGCGCGATGAGACCAAGGGTTTCTGAGACGAAGCCAAGCGGTTCAAGGATACGGCTGGTCAATACGCTACAGCTGCTGGGAACTCTGCTTCCGCTGCGAATCAATCGGGGGGAAACGCTGAGAACTCTGCCACAGCATCCGCTAACTGTGCTCATTTGGCAGAACAGCAAGCAGACCGTGCGGAACGTGAGGCAGACAAGCTGGAAAATTACAATGGATTGGCTGGTGCAATTGATAAGGTAGATAGAACCAATGTGTACTGGAAAGGAAATAtt                                                                                                                                                                              <  1:538511/300‑1 (MQ=255)`  
                                                                                                                               `aaCCAACGCTACGAACACAAAACAGTGGCTCGATGAGACCAAGGGTTTCCGAGACGAAGCCAAGCGGTTCAAGAATACGGCTGGTCAATACGCTACATCTGCTGGGAACTCTGCTTCCGCTGCGCATCAATCTGAGGTAAACGCTGAGAACTTTGCCACAGCATCCGCTAACTATGCTCATTTGGCAGAACAGCAAGCAGACCGTGCGGAACGTGAGGCAGACAAGCTGGAAAATTACAATGGATTGGCTGGTGCAATTGATAAGGTAGATGGAACCAATGTGTACTGGAAAGGAAATAtt                                                                                                                                                                              <  1:584668/300‑1 (MQ=255)`  
                                                                                                                                `tgtgtagaagagacagaaaaGCAGGGGCGCGATGAGACCAAGGGTTTCCGAGACGAAGCCGAGCGGTTCAAGAATACGGCTGGTCAATACGCTACATCTGCTGGGAACTCTGCTTCCGCTGCGCATCAATCTGATGTAAACGCTGAGAACTCTGCCACAGCAGCCGCTAACTCTGCTCATTTGGCAGAACAGCAAGCAGACCGTGCGGAACGTGAGGCAGACAAGCTGGAAAATTACAATGGATTGGCTGGTGCAATTGATAAGGTAGATGGAACCAATGTGTACTGGAAAGGAAATATTc                                                                                                                                                                             <  1:263350/284‑1 (MQ=255)`  
                                                                                                                                     `cGCTACGAACACAAAGCAGTGGCGCGATGAGACCAAGGGTTTCCGAGACGAAGCCAAGCGGTTCAAGAATACGGCTGGTCAATACGCTACATCTGCTGGGAACTCTGCTTCCGCTGCGCATCAATCTGAGGTAAACGCTGAGAACTCTGCCACAGCATCCGCTAACTATGCTCATTTGGCAGCACAGCAAGCAGACCGTGCGGAACGTGAGGAAGACAAGCTGGAAAATTACAATGGATTGGCTGGTGCAATTGATAAGGTAGATGGAACCAATGTGTACTGGAAAGGAAATATTCACGCt                                                                                                                                                                        >  1:466109/1‑301 (MQ=255)`  
                                                                                                                                        `tACGAACACAAAGCAGTGGCGCGATGAGACCAAGGGTTTCCGAGACGAAGCCGAGCGGTTCAAGAATACGGCTGGTCAATACGCTACATCTGCTGGGAACTCTGCTTCCGCTGCGCATCAATCTGAGGTAAACGCTGAGAACTCTGCCACAGCAGCCGCTAACTCTGCTCATTTGGCAGAACAGCAAGCAGACCGTGCGGAACGTGAGGCAGACAAGCTGGAAAATTACAATGGATTGGCTGGTGCAATTGATAAGGTAGATGGAACCAATGTGTACTGGAAAGGAAATATTCACGCTaaa                                                                                                                                                                     >  1:64843/1‑300 (MQ=255)`  
                                                                                                                                              `cacaAAGCAGTGGCGCGATGAGACCAAGGGTTTCCGAGACGAAGCCAAGCGGTTCAAGGATACGGCTGGTCAATACGCTACATCTGCTGGGAACTCTGCTTCCGCTGCGCATCAATCTGAGGTAAACGCTGAGAACTCTGCCACAGCATCCGCTAACTTTGCTCATTTGGCAGAACAGCAAGCAGACCGTGCGGAACGTAAGGCAGACAAGCTGGAAAATTACAATGGATTGGCTGGTGCAATTGATAAGGTAGATGGAACCAATGTGTACTGGAAAGGAAATATTCACGCTAACGGgcgg                                                                                                                                                               >  1:563194/1‑300 (MQ=255)`  
                                                                                                                                              `ataagagacagggCGCGATGAGACCAAGGGTTTCCGAGACGAAGCCAAGCGGTTCAAGGATACGGCTGGTCAATACGCTACATCTGCTGGGAACTCTGCTTCCGCTGCGCATCAATCTGAGGTAAACGCTGAGAACGCTGCCACAGCATCCGCTAACTATGCTCATTTGGCAGAACAGCAAGCAGACCGTGCGGAACGTGAGGCAGACAAGCTGGAAAATTACAATGGATTGGCTGGTGCAATTGATAAGGTAGATGGAACCAATGTGTACTGGAAAGGAAATATTCACGCTAACGGgcgc                                                                                                                                                               <  1:388650/290‑1 (MQ=255)`  
                                                                                                                                                     `cAGTGGCGCGATGAGAGCAAGGGTTTCCGAGACGAAGCCAAGCGGTTCAAGAATACGGCTGGTCAATACGCTACATCTGCTGGGAACTCTGCTTCCGCTGCGAATCAATCTGAGGTAAACGCTGAGAACTCTGCCACAGCATCCGCTAACTATGCTCATTTGGCAGAACAGCAAGCAGACCGTGCGAAACGTGAGGCAGACAAGCTGGAAAATTACAATGGATTGGCTGGTGCAATTGATAAGGTAGATGGAACCAATGTGTACTGGAAAGGAAATATTCACGCTAACGGGCGCCTTTACa                                                                                                                                                        <  1:661015/301‑1 (MQ=255)`  
                                                                                                                                                       `ggatcaggATGAGACCAAGGGTTTCCGAGACGAAGCCAAGCGGTTCAAGGAGACGGCTAGTCAATACGATACATCTGCTGGGAACTCTGCTTCCGCTGCGCATCAATCTGAGGTAAACGCTGAGAACTCTGCCATAGCATCCGCTAACTATGCTCATTTGGCAGAACAGCAAGCAGACCGTGCGGAACTAGAGGCAGACAAGCTGGAAAATTACAATGGATTGGCTGGTGCAATTGATAAGGTAGATGGAACCAATGTGTACTGGAAAGGAAATATTCACGCTAACGGGCGCCTTTACATg                                                                                                                                                      <  1:87445/294‑1 (MQ=255)`  
                                                                                                                                                                   `gaCCAAGGGTTTCCGAGACGAAGCCAATCGGTTCAAGGATACGGCTGGTCAATACGCTACATCTGCTGGGAACTCTGCTTCCGCTGCGCATCAATCTGAGGTAAACGCTGAGAACTCTGCCACAGCATCCGCTAACTTTGCTCATTTGGCAGAACAGCAAGCAGACCGTGCGGAACGTGAGGCAGACAAGCTGGAAAATTACAATGGATTGGCTGGTGCAATTGATAAGGTAGATGGAACCAATGTGTACTGGAAAGGAAATATTCACGCTAACGGGCGCCTTTACATGACAACAAACggg                                                                                                                                          >  1:280174/1‑300 (MQ=255)`  
                                                                                                                                                                   `gaCCAAGGGTTTCCGAGACGAAGCCAAGCGGTTCAAGGATACGGCTGGTCAATACGCTACATCTGCTGGGAACTCTGCTTCCGCTGCGCATCAATCTGAGGTAAACGCTGAGAACTCTGCCACAGCATCCGCTAACTATGCTCATTTGGCAGAACAGCAAGCAGACCGTGCGGAACGTGAGGCAGACAAGCTGGAAAATTACAATGGATTGGCTGGTGCAATTGATAAGGTAGATGGAACCAATGTGTACTGGAAAGGAAATATTCACGCTAACGGGCGCCTTTACATGACCACAAACGGt                                                                                                                                          >  1:478039/1‑301 (MQ=255)`  
                                                                                                                                                                   `gaCCAAGGGTTTCCGAGACGAAGCCAAGCGGTTCAAGAATACGGCTGGTCAATACGCTACATCTGCTGGGAACTCTGCTTCCGCTGCGCATCAATCTGAGGTAAACGCTGAGAACTCTGCCACAGCATCCGCTAACTATGCTCATTTGGCAGAACAGCAAGCATACCGTGCGGAACGTGAGGCAGACAAGCTGGAAAATTACAATGGATTGGCTGGTGCAATTGATAAGGTAGATGGAACCAATGTGTACTGGAAAGGAAATATTCACGCTAACGGGCGCCTTTACATGACCACAAACGGt                                                                                                                                          <  1:568497/301‑1 (MQ=255)`  
                                                                                                                                                                   `gaCCAAGGGTTTCCGAGACGAAGCCAAGCGGTTCAAGAATACGGCTGGTCAATACGCTACATCTGCTGGGAACTCTGCTTCCGCTGCGCATCAATCTGAGGTAAACGCTGAGAACTCTGCCACAGCATCCGCTAACTATGCTCATTTGGCAGAACAGCAAGCAGACCGTGCGGAACGTGAGGCAGACAAGCTGGAAAATTACAATGGATTGGCTGGTGCAATTGATAAGGTAGATGGAACCAATGTGTACTGGAAAGGAAATATTCACGCTAACGGGCGCCTTTACATGACCACAAACGGt                                                                                                                                          >  1:178400/1‑301 (MQ=255)`  
                                                                                                                                                                         `gggTTTCCGAGACGAAGCCAAGCGGTTCAAGAATACGGCTGGTCAATACGCTACATCTGCTGGGAACTCTGCTTCCGCTGCGCATCAATCTGAGGTAAACGCTGAGAACGCTGCCACAGCATCCGCTAGCTATGCTCATTTGGCAGAACAGCAAGCAGACCGTGCGGAACGTGAGGCAGACAAGCTGGAAAATTACAATGGATTGGCTGGTGCAATTGATAAGGTAGATGGAACCAATGTGTACTGGCAAGGAAATATTCACGCTAACGGGCGCCTTTACATGACCACAAACGGTTTTGAc                                                                                                                                    >  1:112253/1‑301 (MQ=255)`  
                                                                                                                                                                          `ggTTTCCGAGACGAAGCCAAGCGGTTCAAGGATACGGCTGGTCAATACGCTACATCTGCTGGGAACTCTGCTTCCGCTGAGCATCAATCTGAGGTAAACGCTGAGAACTCTGCCACAGCATCCGCTAACTATGCTCATTTGGCAGAACAGCAAGCAGACCGTGCGGAACGTGAGGCAGACAAGCTGGAAAATTACAATGGATTGGCTGGTGCAATTGATAAGGTAGATGGAACCAATGTGTACTGGAAAGGAAATATTCACGCTAACTGGCGCCTTTACATGACCACAAACGGTTTTGACt                                                                                                                                   >  1:67516/1‑301 (MQ=255)`  
                                                                                                                                                                                         `tgtataagatgcaggAATACGTCTGGTCAATTCGCTACATCTGCTGGGAACTCTGCTTCCGCTGCGCATCAAACTGAGGTAAACGCTGAGAACTATGCCACAGCATCCGCTAACTATGCTCATTTGGCAGAACAGCAAGCAGACCGTGCGGAACGTGAGGCAGACAAGCTGGAAAATTACAATGGATTTGCTGGTGCAATTGATAAGGTAGATGGAACCAATGTGTACTGGAAAGGAAATATTCACGCTAACGGGCGCCTTTACATGACCACAAACGGTTTTGACTGTGGCCAGTATCAAc                                                                                                                    <  1:130665/287‑1 (MQ=255)`  
                                                                                                                                                                                         `gCCAAGCGGTTCAAGGATACGGCTGGTCAATACGCTACATCTGCTGGGAACTCTGCTTCCGCTGCGCATCAATCTGAGGTAAACGCTTAGAACTCTGCCACAGCATCCGCTAACTTTGCTCATTTGGCAGAACAGCAAGCAGACCGTGCGGAACGTGAGGCAGACAAGCTGGAAAATTACAATGGATTGGCTGGTGCAATTGATAAGGTAGATGGAACCAATGTGTACTGGAAAGGAAATATTCACGCTAACGGGCGCCTTTACATGACCACAAACGGTTTTGACTGTGGCCAGTATCAAc                                                                                                                    <  1:557158/301‑1 (MQ=255)`  
                                                                                                                                                                                         `agggacagggtCAAGGATACCGCTGGTCAATCCGCTACATCTGCCGGGAACTCTTCTTCCGCTGCGCATCAATCTGAGGTAAACGCTGAGAACTCTGCCACAGCATCCGCTAACTTTGCTCATTTGGCAAAACAGCAAGCAGACCGTTCGGAACGTGAGGCAGACAAGCTGGAAAATTACAATGGATTGGCTGGTGCAATTGATAAGGTAGATGGAACCAATGTTTACTGGAAAGGAAATATTCACGCTAACGGGCGCCTTTACATGACCACAAACGGTTTTGACTGTGGCCAGTATCAAc                                                                                                                    <  1:162726/291‑1 (MQ=255)`  
                                                                                                                                                                                           `aaaGCGGTTAAAGAATACGGCTGGTCAATAAGCTACATCGGCTGGGAACACTGCTTCCGATGCGCATCAATCTGAGGTAAACGCTGAGAACTCTGCCAAAGAATCCGCTAACTATGCTCATTTGGCAGAACAGAAAGCAGACCGTGCGGAACGTGAGGCAGACAAGCTGGAAAATTACAATGGATTGGCTGGTGCAATTGATAAGGTAGATGGAACCAATGTGTACTGGAAAGGAAATATTCACGCTAACGGGCGCCTTTACCTGACCACAAACGGTTTTGACTGTGGCCAGTATCAACAg                                                                                                                  <  1:619406/300‑1 (MQ=255)`  
                                                                                                                                                                                                    `cAAGAATACGGCTGGTCAATACGCTACATCTGCTGGGAACTCTGCTTCCGCTGCGCATCAATCTGAGGTAAACGCTGAGAACTCTGCCACAGCATCCGCTAACTATGCTCATTTGGCAGAACAGCAAGCAGACCGTGCGGAACGTGAGGCAGACAAGCTGGAAAATTACAATGGATTGGCTGGTGCAATTGATAAGGTAGATGGAACCAATGTGTACTGGAAAGGAAATATTCACGCTAACGGGCGCCTTTACATGACCACAAACGGTTTTGACTGTGGCCAGTATCAACAGTTCTTtggt                                                                                                         >  1:269409/1‑301 (MQ=255)`  
                                                                                                                                                                                                    `cAAGAATACGGCTGGTCAATACGCTACATCTGCTGGGAACTCTGCTTCCGCTGCGCATCAATCTGAGGTAAACGCTGAGAACTCTGCCACAGCATCCGCTAACTATGCCCATTTGGCAGTACAGCAAGCAGACCGTGCGGAACGTGAGGCAGACAAGCTGGAAAATTACACTGGATTGGCTGGTGCAATTGATAAGGTAGATGGAACCACTGTGTACTGGAAAGGAAATATTCACGCTAACGGGCGCCTTTACATGACCACAAACGGCTTTGAcctgtagcttctacacatctacgagccc                                                                                                         >  1:410629/1‑274 (MQ=255)`  
                                                                                                                                                                                                      `aGAATACGGCTGGTCAATACGCTACATCTGCTGGGAACTCTGCTTCCGCTGCGCATCAATCTGAGGTAAACGCTGAGAACTCTGCCACAGCATCCGCTAACTATGCTCATTTGGCAGAACAGCAAGCAGACCGTGCGGAACGTGAGGCAGACAAGCTGGAAAATTACAATGGATTGGCTGGTGCAATTGATACGGTAGATGGAACCAATGTGTACTGGAAATGCAATATTCACGCTAACGGGCGCCTTTACATGACCACAAACGGTTTTGACTGTGGCCAGTATCAACAGTTCTTtggtgg                                                                                                       >  1:415986/1‑301 (MQ=255)`  
                                                                                                                                                                                                       `ggaTACGGCTGGTCAATACGCTACATCTGCTGGGAACTCTGCTTCCGCTGCGCATCAATCTGAGGTAAACGCTGAGAACTCTGCCACAGCATCCGCTAACTTTGCTCATTTGGCAGAACAGCAAGCAGACCGTGCGGAACGTGAGGCAGACAAGCTGGAAAATTACAATGGATTGGCTGGTGCAATTGATAAGGTAGATGGAACCACTTTGTACTGGAAAGGAAATATTCACGCTAACGGTCGCCTTTACATGACCACAAACGGTTTTGACTGTGGCCAGTCTCTACAGTTCTTTggtggt                                                                                                      >  1:376073/3‑301 (MQ=255)`  
                                                                                                                                                                                                        `aaTACGGCTGGTCAATACGCTACATCTGCTGGGAACTCTGCTTCCGCTGCGCATCAATCTGGGGTAAACTCTGAGAACTCTGCCACAGCATCCGCTAACTTTGCTCATTTGGCAGAACAGCAAGCAGACCGTGCGGAACGTGAGGCAGCCAAGCTGGAAAATTACAATGGATTGGCTGGTGCAATTGATAAGGTAGATGGAACCAATGTGTACTGGAAAGGAAATATTCACGCTAACGGGCGCCTTTACATGACCACAAACGGTTTTGACTGTGGCCAGTATCAACAGTTCTTTGgtggtg                                                                                                     <  1:533450/301‑1 (MQ=255)`  
                                                                                                                                                                                                                 `ggTCAATACGCTACACCTGCTGGGAACTCTGCTTCCGCTGCGCATCAATCTGAGGTAAACGCTGAGAACTCTGCCACAGCATCCGCTAACTATGCTCATTTGGCAGAACAGCAAGCAGACCGTTCGGTACGTGAGGCAGACAAGCTGGAAAATTACAATGGATTGGCTGGTGCAATTGATAAGGTAGATGGAACCAATGTGTACTGGAAAGGAAATATTCACGCTAACGGGCGCCTTTACATGACCACAAACGGTTTTGACTGTGGCCAGTATATACAGTTCTTTGGTGGTGTCACTAATc                                                                                            >  1:437229/1‑301 (MQ=255)`  
                                                                                                                                                                                                                      `aTACGCTACATCTGCTGGGAACTCTGCTTCCGCTGCGCATCAATCTGAGGTAAACGCTGAGAACTCTGCCACAGCATCCGCTAACTATGCTCATTTGGCAGAACAGCAAGCAGACCGTGCGGAACGTGAGGCAGACAAGCTGGAAAATTACAATGGATTGGCTGGTGCAATTGATAAGGTAGATGGAACCAATGTGTACTGGAAAGGAAATATTCACGCTAACGGGCGCCTTTACATGACCACAAACGGTTTTGACTGTGGCCAGTATCAACAGTTCTTTGGTGGTGTCACTAATCGTTAc                                                                                       >  1:213639/1‑301 (MQ=255)`  
                                                                                                                                                                                                                        `ataagagacaggctgGGAACTCTGCTTCCGCTGCGCATCAATCTGAGGTAAACGCTGAGAACTCTGCCACAGCATCCGCTAACTTTGCTCATTTGGCAGAACAGCAAGCAGACCGTGCGGAACGTGAGGCAGACAAGCTGGAAAATTACAATGGATTGGCTGGTGCAATTGATAAGGTAGATGGAACCAATGTGTACTGGAAAGGAAATATTCACGCTAACGGGCGCCTTTACATGACCACAACCGGTTTTGACTGTGGCCAGTATCAACAGTTCTTTGGTGGTGTCACTAATCGTTActc                                                                                     <  1:168159/290‑1 (MQ=255)`  
                                                                                                                                                                                                                        `aCGCTACATCTGCTGGGAACTCTGCTTCCGCTGCGCATCAATCTGAGGTAAACGCTGAGAACTCTGCCACAGCATCCGCTAACTATGCTCAGTTGGAAGAACAGCAAGCAGACCGTGCGGAACGTGAGACAGACAAGCTGGTAAAGTACAATGGATTGGCTGGTGCCATTGATAAGGTAGATGGAGCCAATGTGTACTGGAAAGGAAATATTCACGCTAACGGGCTCCTTTACATGACCACAAACGGTTTTGACTGTGGCCAGTATCAACAGTTCTTTGGTGGTGTCACTAATCGTTActc                                                                                     <  1:370992/301‑1 (MQ=255)`  
                                                                                                                                                                                                                        `aCGCTACATCTGCTGGGAACTCTGCTTCCGCTGCGCATCAATCTGAGGTAAACGCTGAGAACTCTGCCAAAGCATCCGCTAACTCTGCTCATTTGGCAGAACAGCAAGCAGACCGTGCGGCACGTGAGGCAGACAAGCTGGAAAATTACAATGGATTGGCTGGTGCAATTGATAAGGTAGATGGAACCAATGTGTACTGGAAAGGAAATATTCACGCTAACGGGCGCCTTTACATGACCACAAACGGTTTTGACTGTGGCCAGTATCAACAGTTCTTTGGTGGTGTCACTAATCGTTActc                                                                                     >  1:361247/1‑301 (MQ=255)`  
                                                                                                                                                                                                                         `cGCTACATCTGCTGGGAACTCTGCTTCCGCTGCGCATCAATCTGAGGTAAACGCTGAGAACTCTGCCACAGCATCCGCTAACTATGCTCATTTGGCAGAACAGCAAGCAGACCGTGCGGAACGTGAGGCAGACAAGCTGGAAAATTACAATGGATTGGCTGGTGCAATTGATAAGGTAGATGGAACCAATGTGTACTGGAAAGGAAATATTCACGCTAACGGGCGCCTTTACATGACCACAAACGGTTTTGACTGTGGCCAGTATCAACAGTTCTTTGGTGGTGTCACTAATCGTTActct                                                                                    >  1:573785/1‑301 (MQ=255)`  
                                                                                                                                                                                                                                  `tgctgGGAACTCTGCTTCCGCTGCGCATCAATCTGAGGTAAACGCTGAGAACTCTGCCACAGCATCCGCTAACTATGCTCATTTGGCAGAACAGCAAGCAGACCGTGCGGAACGTGAGGCAGACAAGCTGGAAAATTACAATGGATTGGCTGGTGCAATTGATAAGGTAGATGGAACCAATGTGTACTGGAAAGGAAATATTCACGCTAACGGGCGCCTTTACATGACCACAAACGGTTTTGACTGTGGCCAGTATCAACAGTTCTTTGGTGGTGTCACTAATCGTTACTCTGTCATGGAg                                                                           <  1:336695/301‑1 (MQ=255)`  
                                                                                                                                                                                                                                   `gctgGGAACTCTGCTTCCGCTGCGCATCAATCTGAGGTAAACGCTGAGAACTCTGCCACAGCATCCGCTAACTATGCTCATTTGGCAGAACAGCAAGCAGACCGTGCGGAACGTGAGGCAGACAAGCTGGAAAATTACAATGGATTGGCTGGTGCAATTGATAAGGTAGATGGAACCAATGTGTACTGGAAAGGAAATATTCACGCTAACGGGCGCCTTTACATGACCACAAACGGTTTTGACTGTGGCCAGTATCAACAGTTCTTTGGTGGTGTCACTAATCGTTACTCTGTCATGGAGt                                                                          >  1:237264/1‑301 (MQ=255)`  
                                                                                                                                                                                                                                       `atgtgtataagagacaggcgcATCAATCTGAGGTAAACGCTGAGAACTCTGCCACAGCATCCGCTAACTTTGCTCATTTGGCAGAACAGCAAGCAGACCGTGCGGAACGTGAGGCAGACAAGCTGGAAAATTACAATGGATTGGCTGGTGCAATTGATAAGGTAGATGGAACCAATGTGTACTGGAAAGGAAATATTCACGCTAACGGGCGCCTTTACATGACCACAAACGGTTTTGACTGTGGCCAGTATCAACAGTTCTTTGGTGGTGTCACTAATCGTTACTCTGTCATGGAGTgggg                                                                      <  1:488926/284‑1 (MQ=255)`  
                                                                                                                                                                                                                                              `tGCGTCCGCTGCGCATCAATCTGAGGTAAACGCTGAGAACTCTGCCACAGCATCCGCTAACTATGCTCATTTGGCAGAACAGCAAGCAGACCGTGCGGAACGTGAGGCAGACAAGCTGGAAAATTACAATGGATTGGCTGGTGCAATTGATAAGGTAGATGGAACCAATGTGTACTGGAAAGGAAATATTCACGCTAACGGGAGCCTTTACATGACCACAAACGGTTTTGACTGTGGCCAGTATCAACAGTTCTTTGGTGGTGTCACTAATCGTTACTCTGTCATGGAGTGGGGAGATgag                                                               <  1:353168/301‑1 (MQ=255)`  
                                                                                                                                                                                                                                                          `gcATCAAACGGAGGTAAACGATGAGAACGCTGCCACAGCATCCGCTAACTGTGATCATCTGGCAGAACAGCAAGAAGACCGTGCGGAACGTGAGGCAGACCAGCTGGAAAATTACAATGGATTGGCTGGTGCAATTGATAAGGTATATGGAACCCATGTGTACTGGAAAGGAAATATTCACGCTAACGGGCGCCTTTACATGACCACAAACGGTTTTGACTGTGGCCAGTATCAACAGTTCTTTGGTGGTGTCACTAATCGTTACTCTGTCATGGAGTGGGGAGATGAGAACGGATGGCTg                                                   <  1:455310/301‑1 (MQ=255)`  
                                                                                                                                                                                                                                                              `aaaTCTGAGGTAAACGCTGAGAACTCTGCCACAGCATCCGCTAACTATGCTCATTTGGCAGAACAGCAAGCAGACCGTGCGGAACGTGAGGCAGACAAGCTGGAAAATTACAATGGATTGGCTGGTGCAATTGATAAGGTAGATGGAACCAATGTGTACTGGAAAGGAAATATTCACGCTAACGGGCGCCTTTACATGACCACAAACGGTTTTGACTGTGGCCAGTATCAACAGTTCTTTGGTGGTGTCACTAATCGTTACTCTGTCATGGAGTGGGGAGATGAGAACGGATGGCTGatgt                                               <  1:173233/300‑1 (MQ=255)`  
                                                                                                                                                                                                                                                               `aaTCTGAGGTAAACGCTGAGAACTTTGCCACAGCATCCGCTAACGATGCTCATTTGGCAGAACAGCAAGCAGACCGTGCGGAACGTGAGGCAGACAAGCTGGAAAATTACAATGGATTGGCTGGTGCAATTGATAAGGTAGATGGAACCAATGTGTACTGGAAAGGAAATATTCACGCTAACGGGCGCCTTTACATGACCACAAACGGTTTTGACTGTGGCCAGTATCAACAGTTCTTTGGTGGTGTCACTAATCGTTACTCTGTCATGGAGTGGGGAGATGAGAACGGATGGCTGatgta                                              <  1:625378/301‑1 (MQ=255)`  
                                                                                                                                                                                                                                                               `aaTCTGAGGTAAACGCTGAGAACTCTGCCACAGCATCCGCTAACTATGCTCATTTGGCAGAACAGCAAGCAGACCGTGCGGAACGTGAGGCAGACAAGCTGGAAAATTACAATGGATTGGCTGGTGCAATTGATAAGGTAGATGGAACCAATGTGTACTGGAAAGGAAATATTCACGCTAACGGGCGCCTTTACCTGACCACAAACGGTTTTGACTGTGGCCAGTATCAACAGTTCTTTGGTGGTGTCACTAATCGTTACTCTGTCATGGAGTGGGGAGATGAGAACGGATGGCTGatgta                                              <  1:256270/301‑1 (MQ=255)`  
                                                                                                                                                                                                                                                                    `gAGGTACACGCTGAGAACTCGACCACAGCATCCGCTAACTATGCTCATTTGGCAGAACAGCAAGCAGACCGGGCGGAACGTGAGGCAGACAAGCTGGAAAATTACAATGGATTGGCTGGTGCAATTGATAAGGTAGATGGAACCAATGTGTACTGGAAAGGAAATATTCACGCTAACGGGCGCCTTTACATGACCACAAACGGTTTTGACTGTGGCCAGTATCAACAGTTCTTTGGTGGTGTCACTAATCGTTACTCTGTCATGGAGTGGGGAGATGAGAACGGATGGCTGATGTATGTTc                                         <  1:600191/301‑1 (MQ=255)`  
                                                                                                                                                                                                                                                                    `gAGGTAAACGCTGAGAACTTTGCCACAGCATCCGCTAACTATGCTCATTTGGCAGAACAGCAAGCAGACCGTGCGGAACGTGAGGCAGACAAGCTGGAAAATTACAATGGATTGGCTGGTGCAATTGATAAGGTAGATGGAACCAATGTGTACTGGAAAGGAAATATTCACGCTAACGGGCGCCTTTACATGACCACAAACGGTTTTGACTGTGGCCAGTATAAACAGGTCTTTGGTGGTGTCACTAATAGTTACTCTGTCATGGAGTGGGGAGATGAGAACGGATGGCTGATGTATGTTc                                         >  1:50629/1‑301 (MQ=255)`  
                                                                                                                                                                                                                                                                    `gAGGTAAACGCTGAGAACTCTGCCACAGCATCCGCTAACTATGCTCATTTGGCAGAACAGCAAGCAGACCGTGCGGAACGTGAGGCAGACAAGCTGGAAAATTACAATGGATTGTCTGGTGCAATTGATAAGGTAGATGGAACCAATTGGTACTGGAAAGGAAATATTCACGCTAACGGGCGCCTTTACATGACCACAAACGGTTTTGACTGTGGCCAGTATCAACAGTTCTTTGGTGGTGTCACTAATCGTTACTCTGTCATGGAGTGGGGAGATGAGAACGGATGGCTGATGTATGTTc                                         <  1:666044/301‑1 (MQ=255)`  
                                                                                                                                                                                                                                                                    `gAGGTAAACGCTGAGAACTCTGCCACAGCATCCGCTAACTATGCTCATTTGGCAGAACAGCAAGCAGACCGTGCGGAACGTGAGGCAGACAAGCTGGAAAATTACAATGGATTGTCTGGTGCAATTGATAAGGTAGATGGAACCAATGTGTACTGGAAAGGAAATATTCACGCTAACGGGCGCCTTTACCTGACCACAAACGGTTTTGACTGTGGCCAGTATCAACAGTTCTTTGGTGGTGTCACTAATCGTTACTCTGTCATGGAGTGGGGAGATGAGAACGGATTGCTGATGTATGTTc                                         >  1:448601/1‑301 (MQ=255)`  
                                                                                                                                                                                                                                                                      `ggTAAACGCTGAGAACTCTGCCACAGCATCCGCTAACTATGCTCATTTGGCAGAACAGCAAGCAGAACGTGCGGAACGTGAGGCAGACAAGCTGGAAAATTACAATGGATTGGCTGGTGCAATTGATAAGGTAGATGGAACCAATGTGTACTGGAAAGGCAATATTCACGCTAACGGGCGCCTTTACCTGACCACAAACGGTTTTGACTGTGGCCAGTATCAACATTTCTTTGGTGGTGTCACTAATCGTTACTCTGTCATGGAGTGGGGAGATGAGAATGGATGGCTTATGTCTGTTCat                                       >  1:136501/1‑300 (MQ=255)`  
                                                                                                                                                                                                                                                                           `aCGCTGAGAACTCTGCCACAGCATCCGCTAACTATGCTCATTTGGCAGAACAGCAAGCAGACCGTGCGGAACGTGAGGCAGACAAGCTGGAAAATTACAATGGATTGGCTGGTGCAATTGATAAGGTAGATGGAACCAATGTGTACTGGAAAGGAAATATTCACGCTAACGGGCGCCTTTACCTGACCACAAACGGTTTTGACTGTGGCCAGTATCAACAGTTCTTTGGTGGTGTCACTAATCGTTACTCTGTCATGGAGTGGGGAGATGAGAACGGATGGCTGATGTATGTTCAACGTag                                  <  1:470864/301‑1 (MQ=255)`  
                                                                                                                                                                                                                                                                              `cTGAGAACTCTGCCACAGCATCCGCTAACTTTGCTCATTTGGCAGAACAGCAAGCAGACCGTGCGGAACGTGAGGCAGACAAGCTGGAAAATTACAATGGATTGGCTGGTGCAATTGATAAGGTAGATGGAACCAATGTGTACTTGAAAGGAAATATTCACGCTAACGGGCGCCTTTACATGACCACAAACGGTTTTGACTGTGGCCAGTATCAACAGTTCTTTGGTGGTGTCACTAATCGTTACTCTGTCATGGAGTGGGGAGATGAGAACGGATGGCTGATGTATGTTCAACGTagagg                               >  1:590187/1‑300 (MQ=255)`  
                                                                                                                                                                                                                                                                                   `aaCTCTGCCACAGCATCCGCTAACTATGCTCATTTGGCAGAACAGCAAGCAGACCGTGCGGAACGTGAGGCAGACAAGCTGGAAAATTACAATGGATTGGCTGGTTCAATTGATAAGGTAGATGGAACCAATGTGTACTGGAAAGGAAATATTCACGCTAACGGGCGCCTTTACATGACCACAAACGGTTTTGACTGTGGCCCGTATCAACAGTTCTTTGGTGGTGTCCCTAATAGTTACTCTGTCATGGAGTGGGGAGCTGAGAACGGATGGCTGATGTATGTTCCACGTATAGAGTGGa                          >  1:484031/1‑301 (MQ=255)`  
                                                                                                                                                                                                                                                                                     `ctctGCCACAGCATCCGCTAACTTTGCTCATTTGGCAGAACAGCAAGCAGACCGTGCGGAACGTGAGGCAGACAAGCTGGAAAATTACAATGGATTGGCTGGTGCAATTGATAAGGTAGATGGAACCAATGTGTACTGGAAAGGAAATATTCACGCTAACGGGCGCCTTTACATGACCACAAACGGTTTTGACTGTGGCCAGTATCAACAGTTCTTTGGTGGTGTCACTAATCGTTACTCTGTCATGGAGTGGGGAGATGAGAACGGATGGCTGATGTATGTTCAACGTAGATATGGGaca                        >  1:155929/1‑301 (MQ=255)`  
                                                                                                                                                                                                                                                                                         `gCCACAGCATCCGCTAACTTTGCTCATTTGGCAGAAAAGCAAGCAGACCGTGCGGAACGTGAGGAAGACAAGCGGGAAAATTACAATGGATGGGCTGGTGCAATTGATAAGGTAGATGGAACCAATGTGTACTGGAAAGGAAATATTCACGCTAACGGGCGCCTTTACATGACCACAAACGGTTTTGACTGTGGCCAGTATCAACAGTTCTTTGGTGGTGTCACTAATCGTTACTCTGTCATGGAGTGGGGAGATGAGAACGGATGGCTGATGTATGTTCAACGTAGAGAGTGGACAACAg                    <  1:20943/301‑1 (MQ=255)`  
                                                                                                                                                                                                                                                                                         `gCCACAGCATCCGCTAACTATGCTCATTTGGCAGAACAGCAAGCAGACCGTGCGGAACGTGAGGCAGACAAGCTGGAAAATTACAATGGATTGGCTGGTGCAATTGATAAGGTAGATGGAACCAATGTGTACTGGAAAGGAAATATTCACGCTAACGGGCGCCTTTACATGACCACAAACGGTTTTGACTGTGGCCAGTATCAACAGTTCTTTGGTGGTGTCACTAATCGTTACTCTGTCATGGAGTGGGGAGATGAGAACGGATGGCTGATGTATGTTCAACGTAGAGAGTGGACAACAg                    <  1:119863/301‑1 (MQ=255)`  
                                                                                                                                                                                                                                                                                                `aaTCCGCTAACATTGCACAGTTGAGAAAAAAGAAAGCAGACCGTGCGGAACGTGAGGCAGACAAGATGGAAAATTACAATGGATTGGCTGGAGCAATTGAGAAGGTAGATGAAACCAATGTGTACTGGAAAGGAAATATTCACGCTAACGGGCGCCTTTACATGACCAAAAACGGTTGTGACTGTGGCCAGTATCAACAGTTCTTTGGTGGTGTCACTAATCGTTACTCTGTCATGGAGTGGGGAGATGAGAACGGATGGCTGATGTATGTTCAACGTAGAGAGTGGACAACAGCGATAgg             <  1:306831/300‑1 (MQ=255)`  
                                                                                                                                                                                                                                                                                                   `ccGCTAACTTTGCTCATTTGGCAGAACAGCAAGCAGACCGTGCGGAACATGAGGCAGACAAGCTGGAAAATTACAATGGATTGGCTGGTGCAATTGATAAGGTAGATGGAACCAATGTGTACTGGAAAGGAAATATTCACGATAACGGGCGCCTTTACATGACCACAAACGGTTTTGACTGTGGCCAGTATCAACAGTTCTTTGGTGGTGTCACTAATCGTTACTCTGTCATGGAGTGGGGAGATGAGAACGGATGGCTGATGTATGTTCAACGTAGAGAGTGGACAACAGCGATAGGCgg          >  1:331504/1‑301 (MQ=255)`  
                                                                                                                                                                                                                                                                                                       `tAACTATGCTCATTGGGCAGAACAGCAAGCAGACCGTGCGGAACGTGAGGCAGACAAGCTGGAAAATTACAATGGATTGGCTGGTGAAATTGATAAGGTAGATGGAACCAATGTGTACTGGAAAGGAAATATTCACGCTAACGGGCGCCTTTACATGACCACAAACGGTTTTGACTGTGGCCAGTATCAACAGTTCTTTGGTGGTGTCACTAATCGTTACTCTGTCATGGAGTGGGGAGATGAGAACGGATGGCTGATGTATGTTCAACGTAGAGAGTGGACAACAGCGATAGGCGGTAAc      <  1:429856/301‑1 (MQ=255)`  
                                                                                                                                                                                                                                                                                                        `aaCTTTGCTCATTTGGCAGAACAGCAAGCAGACCGTGCGGAACGTGAGGCAGACAAGCTGGAAAATTACAATGGATTGGCTGGTGCAATTGATAAGGTAGATGGAACCAATGTGTACTGGAAAGGAAATATTCACGCTAACGGGCGCCTTTACATGACCACAAACGGTTTTGACTGTGGCCAGTATCAACAGTTCTTTGGTGGTGTCACTAATCGTTACTCTGTCATGGAGTGGGGAGATGAGAACGGATGGCTGATGTATGTTCAACGTAGAGAGTGGACAACAGCGATAGGCGGTAACa     >  1:461127/1‑301 (MQ=255)`  

                                                                                                                                                                                                                                                                                                            `|`                                                                                                                                                                                                                                                                                                             
`CTGTTCCGTTTGGTCAACTAAAGACCATGAACCAGAACTCATGGCAAGCACGTAATGAAGCCTTACAGTTCCGTAATGAGGCTGAGACTTTCAGAAACCAAGCGGAGGGCTTTAAGAACGAGTCCAGTACCAACGCTACGAACACAAAGCAGTGGCGCGATGAGACCAAGGGTTTCCGAGACGAAGCCAAGCGGTTCAAGAATACGGCTGGTCAATACGCTACATCTGCTGGGAACTCTGCTTCCGCTGCGCATCAATCTGAGGTAAACGCTGAGAACTCTGCCACAGCATCCGCTAACTCTGCTCATTTGGCAGAACAGCAAGCAGACCGTGCGGAACGTGAGGCAGACAAGCTGGAAAATTACAATGGATTGGCTGGTGCAATTGATAAGGTAGATGGAACCAATGTGTACTGGAAAGGAAATATTCACGCTAACGGGCGCCTTTACATGACCACAAACGGTTTTGACTGTGGCCAGTATCAACAGTTCTTTGGTGGTGTCACTAATCGTTACTCTGTCATGGAGTGGGGAGATGAGAACGGATGGCTGATGTATGTTCAACGTAGAGAGTGGACAACAGCGATAGGCGGTAACATCC  >  NC_001604.1/35066‑35665`

  

Alignment Legend

Aligned base mismatch/match (shaded by quality score): `ATCG/ATCG` `< 3 ≤` `ATCG/ATCG` `< 10 ≤` `ATCG/ATCG` `< 21 ≤` `ATCG/ATCG` `< 34 ≤` `ATCG/ATCG` `< 38 ≤` `ATCG/ATCG`

Unaligned base: `atcg`    Masked matching base: `atcg`    Alignment gap: `‑`    Deleted base: `‑`
